# Supplementary material for: The mutational and phenotypic spectrum of TUBA1A-associated tubulinopathy
Source: Orphanet J Rare Dis. 2019 Feb 11;14:38. doi: 10.1186/s13023-019-1020-x (PMC6371496; doi:10.1186/s13023-019-1020-x)
Supplement: Supplementary file 1 — Figure S1. Cranial MRI planes of individual i084n. Figure S2. Cranial MRI planes of individual i085n. Figure S3. Cranial MRI planes and clinical pictures of individual i086n. Figure S4. Additional computational scores for TUBA1A variants. Figure S5. Analysis of the variant cluster around amino acid position 400. Figure S6. Comparison of computational scores for TUBA1A variants identified in fetuses and born individuals. Figure S7. Matrix plot of all HPO phenotype categories. Figure S8. Association plots for recurrently affected amino-acid positions and all neuroradiological features. Figure S9. Association plots publications describing ≥ 5 individuals and all neuroradiological features. Table S1. Barthel Index of Activities of Daily Living [3] of the 3 individuals with TUBA1A variants. (DOCX 2946 kb) [file 13023_2019_1020_MOESM1_ESM.docx]

**Supplementary Information:**

**The mutational and phenotypic spectrum of *TUBA1A*-associated tubulinopathy**

Moritz Hebebrand^1^, Ulrike Hüffmeier^1^, Regina Trollmann^2^, Ute Hehr^3^, Steffen Uebe^1^, Arif B. Ekici^1^, Cornelia Kraus^1^, Mandy Krumbiegel^1^, André Reis^1^, Christian T. Thiel^1*#^, Bernt Popp^1#^

^1^Institute of Human Genetics, Friedrich-Alexander-Universität Erlangen-Nürnberg (FAU), Erlangen, Germany

^2^Department of Pediatrics, Division of Neuropediatrics, Friedrich-Alexander-Universität Erlangen-Nürnberg (FAU), Erlangen, Germany

^3^Institute of Human Genetics, University of Regensburg, Regensburg, Germany

# Authors contributed equally to this work

* To whom correspondence should be addressed:

PD Dr. Christian T. Thiel

Institute of Human Genetics

Friedrich-Alexander-Universität Erlangen-Nürnberg (FAU)

Schwabachanlage 10, 91054 Erlangen, Germany

Tel.: +49-9131-85-44637

Email: [Christian.Thiel@uk-erlangen.de](mailto:Christian.Thiel@uk-erlangen.de)

**CLINICAL REPORTS**

Retrospectively collected clinical data of 3 individuals with pathogenic missense variants in *TUBA1A* who presented between 1999 and 2016 at our Center of Developmental Neurology and Social Pediatrics and our genetic clinic. Phenotype terms are used according to the Human Phenotype Ontology (HPO)^1^ where available. Additionally, seizure types are described according to the ILAE 2017 guideline^2^ for epilepsy terminology. Barthel index of activities of daily living scores^3^ were attained for all 3 individuals either by parent reports or recorded observations.

**Individual i084n**: A 13 years and 7 months old boy was the second child of healthy non-consanguineous parents of European descent and was born at term after an uneventful pregnancy. At age six months, the boy initially presented with a focal seizure with impaired awareness. Various electroencephalographic measurements (EEG) showed pathological findings and epilepsy with infantile onset multifocal seizures was suspected. Anticonvulsive therapy with valproic acid was started at the age of 7 months when seizure frequency increased up to more than 10 epileptic spasms per day. Medication with phenobarbital and later co-medication with topiramate resulted in a decreased frequency of epileptic spasms, however only transiently. After initiating a co-medication with vigabatrin and valproic acid a seizure-free period of 12 months was achieved. From age of 2 years until 6 years and 6 months the patient was seizure-free with a monotherapy of valproic acid. Consequently, valproic acid was discontinued under close monitoring without recurrence of seizure activity. Cerebral magnetic resonance imaging (MRI) revealed coarsened cerebral gyri, a hypoplasia of the corpus callosum (HP:0002079), cerebellar vermis hypoplasia (HP:0001320), ventricular dilatation (HP:0002119) and narrowed white matter. Since early infancy, severe generalized muscular hypotonia (HP:0001252) and, global developmental delay (HP:0001263) were present. At the age of 4 years, in addition to global developmental delay, spasticity (HP:0001257) convergent strabismus (HP:0020045, hierarchy HP:0000486), a visual perception disorder and a mild pyelectasis were documented. On referral at the age of 13 years, the boy presented with a weight of 41.0 kg (10th - 25th centile), a height of 146.3 cm (3rd centile) and an occipitofrontal head circumference (OFC) of 52 cm (<3rd centile). Minor facial features included a flat forehead, low set ears, epicanthic fold, upward slanting palpebral fissures, narrow nasal bridge, broad nose tip, short philtrum and an everted lower lip. In addition, a clinodactyly V, a small forefoot and a sandal gap were noticed. He received a Barthel ADL index of 12 / 20 (Table S1). Family history and previous genetic workup including chromosome and chromosomal microarray (CMA) were unremarkable.

**
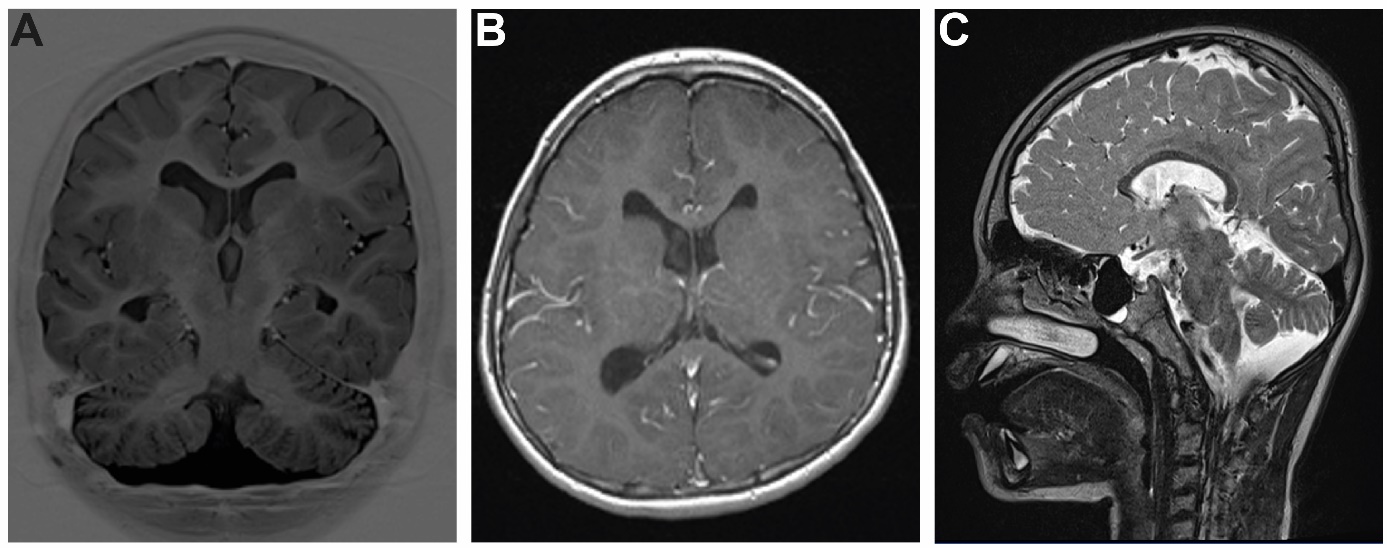
**

**Figure S1 | Cranial MRI planes of individual** **i084n.**

Cranial MRI of individual i084n at the age of 6 years demonstrating coarsened cerebral gyri in the parietal region, a mild hypoplasia of the corpus callosum, ventricular dilatation (A, B), and cerebellar vermis hypoplasia (C). (A) T2Flair, coronal (B) T1w + Gadolinium, transversal (C) T2w, sagittal

**Individual i085n:** A 11 years and 6 months old boy was the second child of healthy non-consanguineous parents of European descent. He was born at term after an uneventful pregnancy with parameters in the normal range [4170 g (85th - 97th centile), 55 cm (97th centile), and OFC 34 cm (10th - 25th centile)]. He had global developmental delay (HP:0001263) and cerebral MRI revealed mild frontal cortical anomalies, a hypoplasia of the corpus callosum (HP:0002079), basal ganglia dysgenesis (HP:0025102), ventricular dilatation (HP:0002119), accentuated lamina quadrigemina and a retrocerebellar arachnoid cyst. The boy presented with a weight of 87.6 kg (>97th centile), height of 172 cm (97th centile) and head circumference of 56 cm (25th - 50th centile). His facial gestalt included a high forehead, large earlobes, epicanthic folds, hypertelorism, jaw deformity, cupid bow shaped upper lip with open mouth appearance, high arched palate and a gap between the upper incisors. Minor features were distally located thumbs, pointed fingers, hallux valgus, and a sandal gap. He received a Barthel ADL index of 11 / 20 (Table S1). Family history and previous genetic testing including CMA were unremarkable.


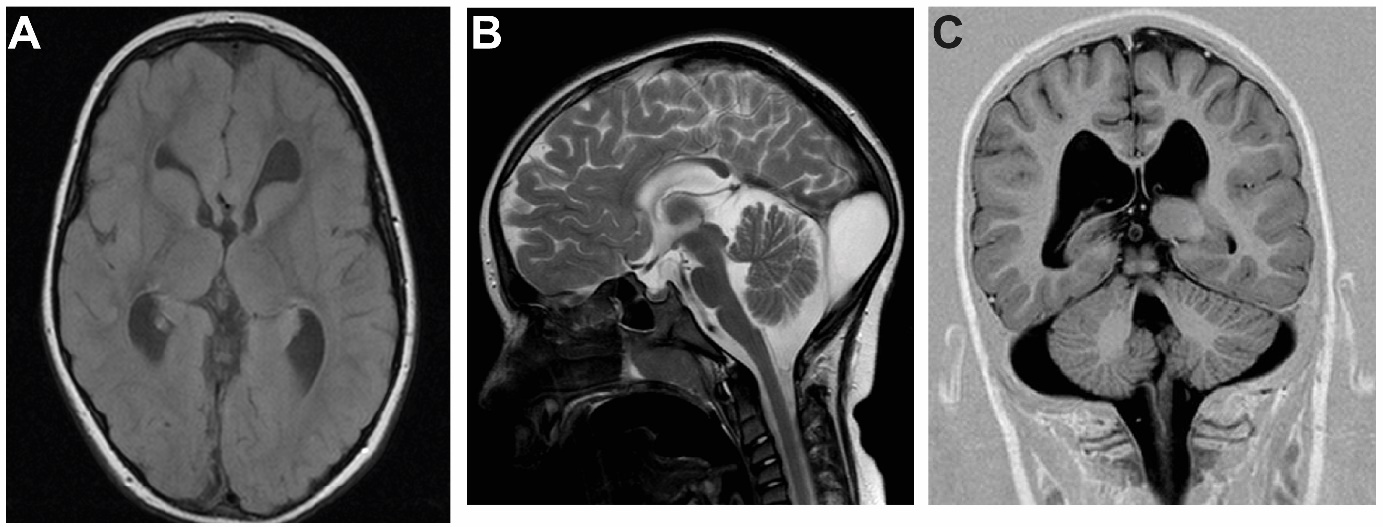


**Figure S2 | Cranial MRI planes of individual** **i085n.**

Cranial MRI of individual i085n with asymmetric frontal and predominantly right sided abnormal cortical gyration, dysmorphic basal ganglia (A), markedly hypoplastic corpus callosum, accentuated lamina quadrigemina, retrocerebellar arachnoid cyst (B) and ventricular dilatation (C).

**Individual i086n:** A 9 years and 3 months old girl was the first child of healthy non-consanguineous parents of European descent. She was born at term with parameters in the normal range [3050 g (15th - 40th centile), 51 cm (75th - 90th centile), OFC 34 cm (25th - 50th centile)]. At age six months, a muscular hypotonia (HP:0001252) and nystagmus (HP:0000639) were noted followed by developmental regression at 1 year 9 months. At age of 1 year and 11 months the girl started to have 5-6 seizures a day with a duration up to 20-30 seconds. After the diagnosis of focal to bilateral tonic-clonic seizures anticonvulsive therapy with valproic acid was initiated, which led to slightly improved EEG findings. When attacks with tonic head turns, behavior arrests and myoclonic seizures re-occurred up to four times a day, lamotrigine was additionally prescribed resulting in a short-term improvement. Due to a subsequent deterioration an anticonvulsive co-medication with valproic acid and topiramate was started. At the age of 8 years and 5 months short lasting episodes of tonic arm extensions up to 60 seconds reoccurred in the morning. Cerebral MRI revealed a Dandy walker variant syndrome, with cerebellar vermis hypoplasia (HP:0001320), agenesis of corpus callosum (HP:0001274) and ventricular dilatation (HP:0002119). Further features were dysgenesis of the basal ganglia (HP:0025102) and unilateral optic nerve hypoplasia (HP:0008058). At last physical examination (age 9 years 3 months), she presented with a height of 125 cm (<3rd centile) and head circumference of 50.5 cm (10th - 25th centile). Global developmental delay (HP:0001266) with absent speech and muscular hypotonia (HP:0001252) with spasticity (HP:0001257) of the legs were noted. Minor facial features included large ears, hypertelorism, broad flat nasal-bridge, high arched palate, wide spaced teeth, thin lips, short neck, smooth philtrum and a simian crease. Additional features included proximal located thumbs, pointed fingers, brachymesophalangia V and clinodactyly V. She received a Barthel ADL index of 0 / 20 (Table S1). Previous testing including CMA was unremarkable. The identification of the *TUBA1A* variant in this girl was part of a previous publication without detailed clinical description (reported as ID S_006).^4^


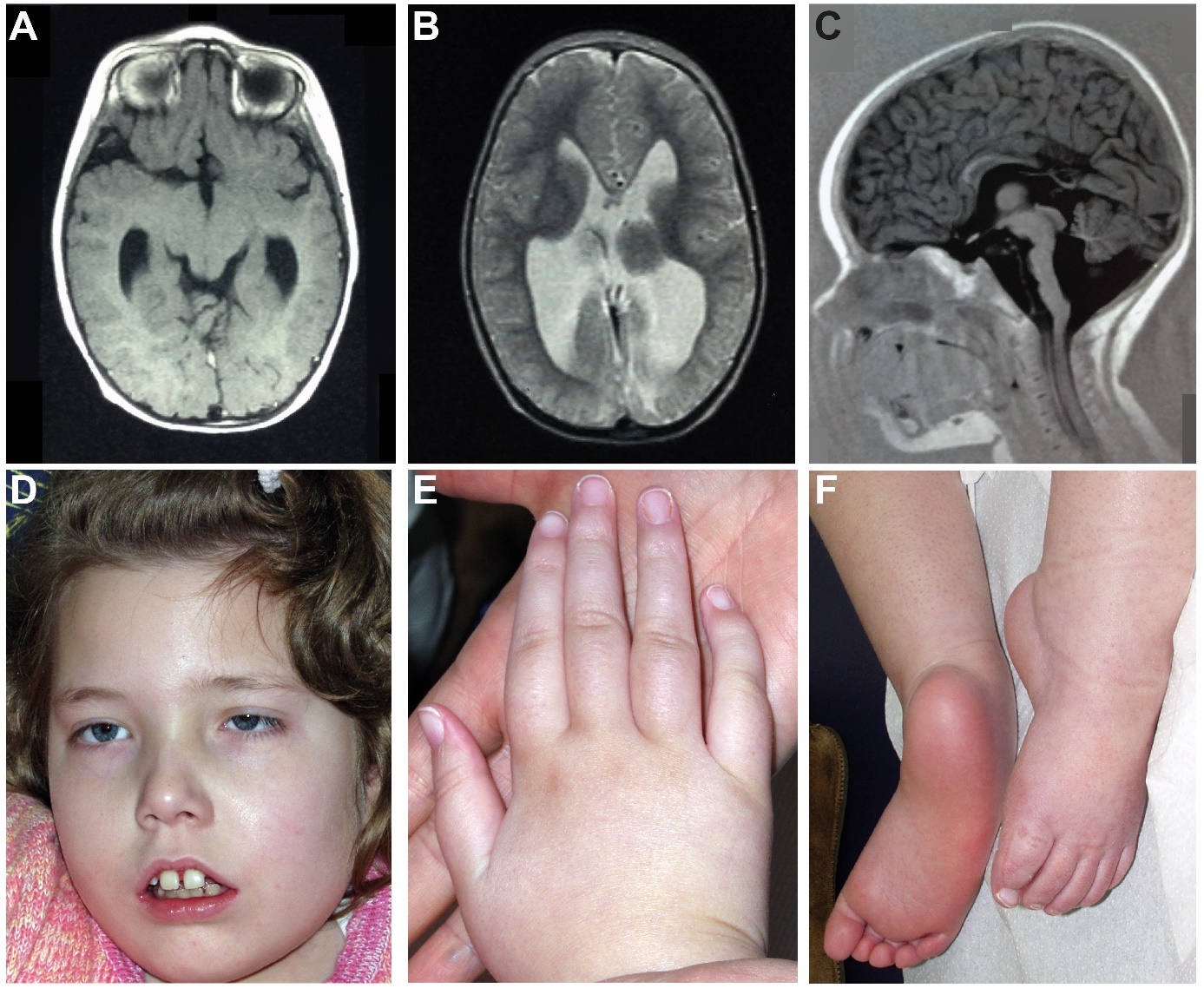


**Figure S3 | Cranial MRI planes and clinical pictures of individual** **i086n.**

(A-C) Cranial MRI (A: T2w, transversal; B: T1w, transversal, C: T1w, sagittal) at the age of 23 months revealed agenesis of corpus callosum and ventricular dilatation (A, B) as well as dysgenesis of the basal ganglia (B) and cerebellar vermis hypoplasia (C) consistent with the diagnosis of Dandy walker variant syndrome. (D) Facial photograph showing hypertelorism, broad flat nasal-bridge, wide spaced teeth, thin cupid bow shaped lips and smooth philtrum. (E) Photograph of the right hand with relatively long, thinning of the distal fingers and clinodactyly V. (F) Photograph of the feet showing clubfoot deformation due to spasticity.

**
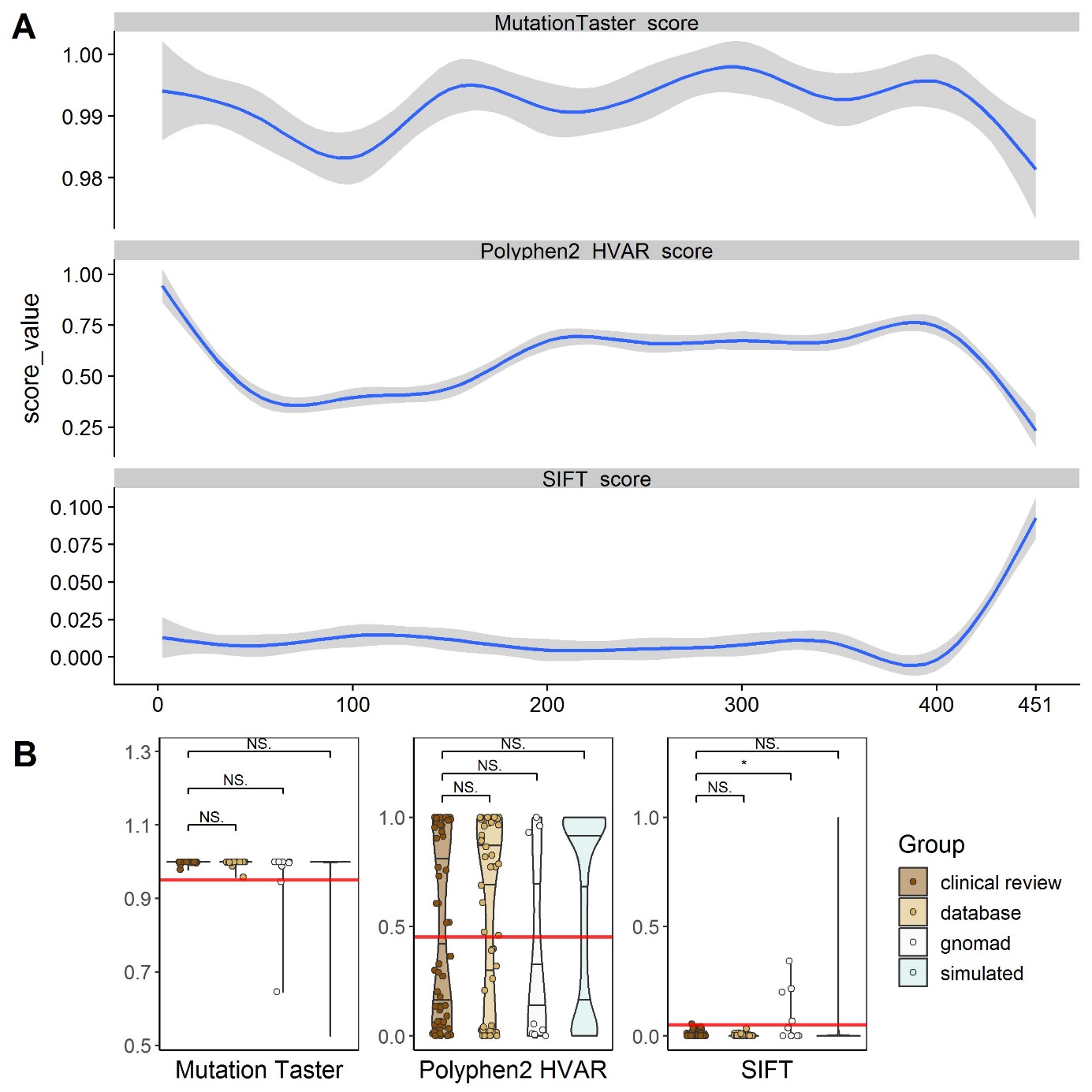
SUPPLEMENTARY RESULTS**

**Figure S4 | Additional computational scores for *TUBA1A* variants.**

(A) Generalized additive models of the MutationTaster, Polyphen2 and SIFT scores for all possible missense variants. (B) Violin- and scatter-plot comparing the MutationTaster, M-Polyphen2 and SIFT computational scores for missense variants in four variant groups. Polyphen2 shows a similar GAM curve like the ensemble scores in main Fig. 1C with a peak around amino-acid position 400. The MutationTaster and SIFT scores in contrast, show a different curve. The violin- and scatter-plot for these three computational scores also mostly showed no significant difference for the four missense variant groups. It should be noted that MutationTaster categorizes variants into boolean groups (disaease causing and not disease causing) and the score represents a confidence into this categorization. Two-sided Wilcoxon signed-rank used for significant testing. NS: not significant, *: P<0.05.


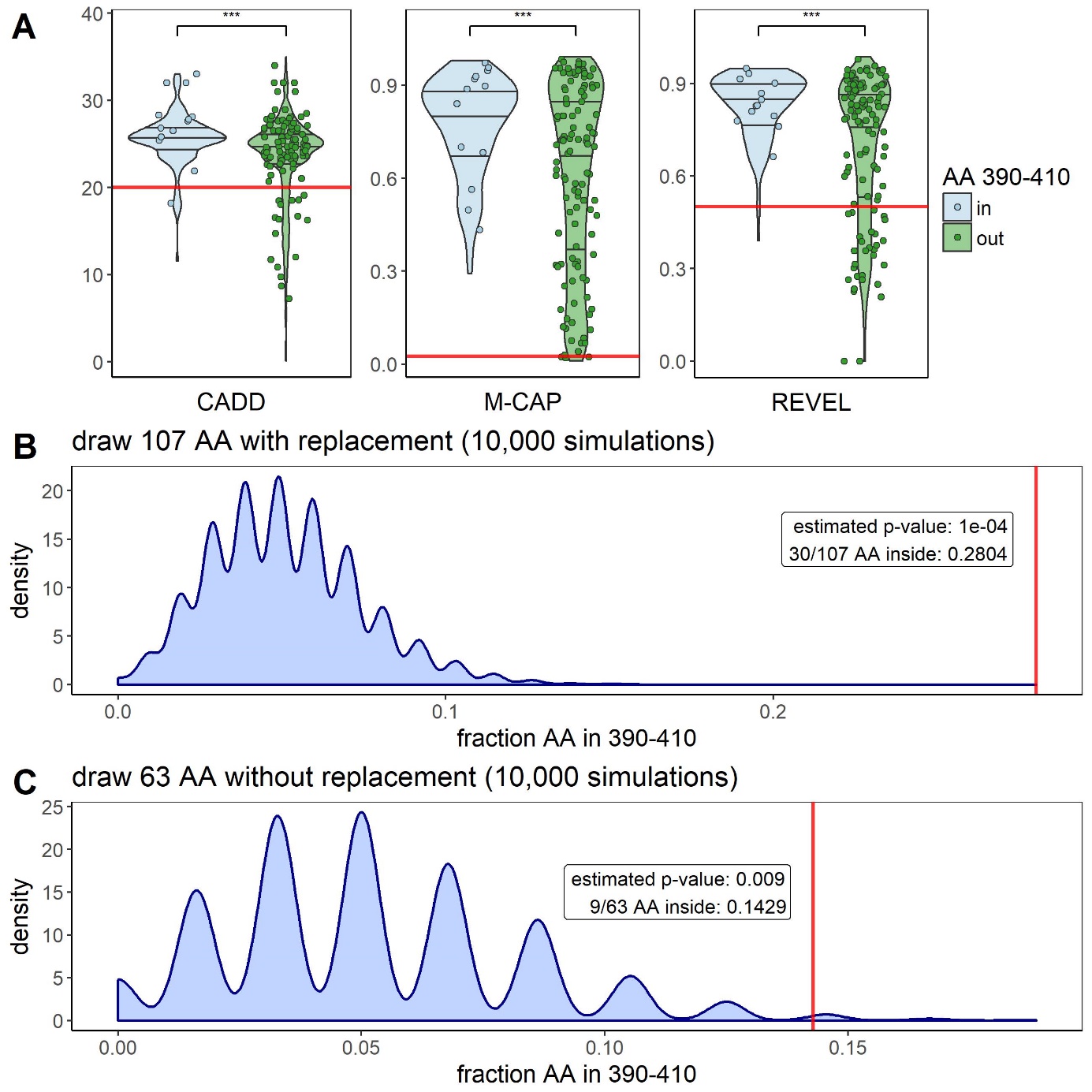


**Figure S5 | Analysis of the variant cluster around amino acid position 400.**

(A) Violin- and scatter-plot comparing the three computational scores CADD, M-CAP and REVEL for missense variants inside (“in”, blue) and outside (“out”, green) the amino acid (AA) region 390-410 of TUBA1A. Two-sided Wilcoxon signed-rank used for significant testing. ***: P<0.001. (B) Empiric distribution for drawing 107 missense variants from all possible 2969 missense substitutions in the AA region 390-410 of TUBA1A with replacement. It is highly unlikely (estimated p-value = (draws ≥ 30/107 in) + 1) / (all draws + 1) ~ 1e^-4^) to draw the observed 30 variants inside this region by chance assuming uniform mutation distribution. (C) Empiric distribution for drawing 63 unique (without replacement) missense variants. This highlights an enrichment (estimated p-value = (draws ≥ 9/63 in) + 1) / (all draws + 1) ~ 0.009) for the 9 observed pathogenic variant positions in the AA 390-410 region even without recurrent variants.


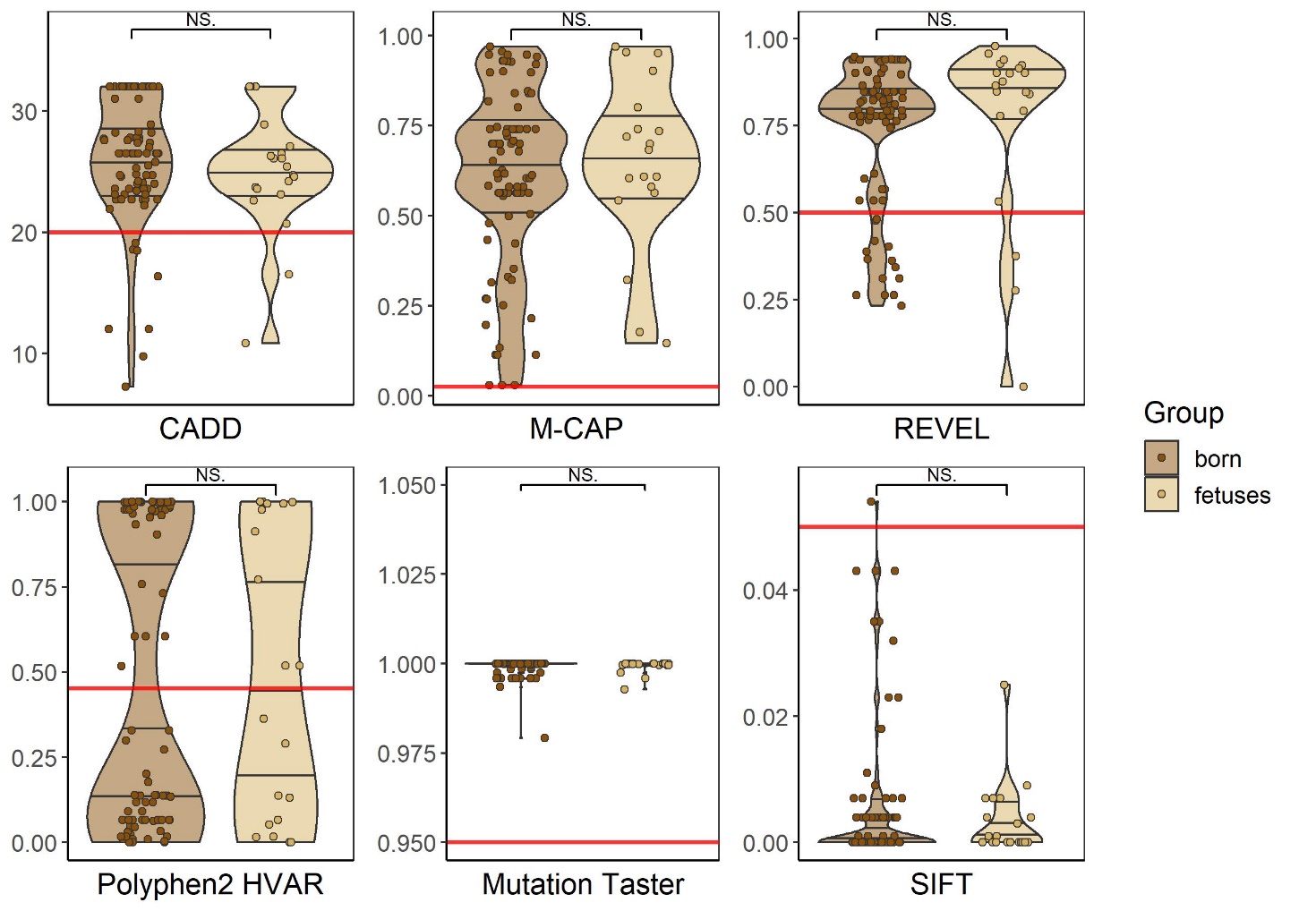


**Figure S6 | Comparison of computational scores for *TUBA1A* variants identified in fetuses and born individuals.**

Missense variants from the review and from the three cases reported here (n=107) show no significant difference in six commonly used computational scores classification of missense variants between fetuses (n=20) and born individuals (n=87). This indicated that properties of missense variants do not explain the observed more severe manifestation in fetuses (“fetuses”) compared to born individuals (“born”). Two-sided Wilcoxon signed-rank used for significant testing. NS: not significant.

**
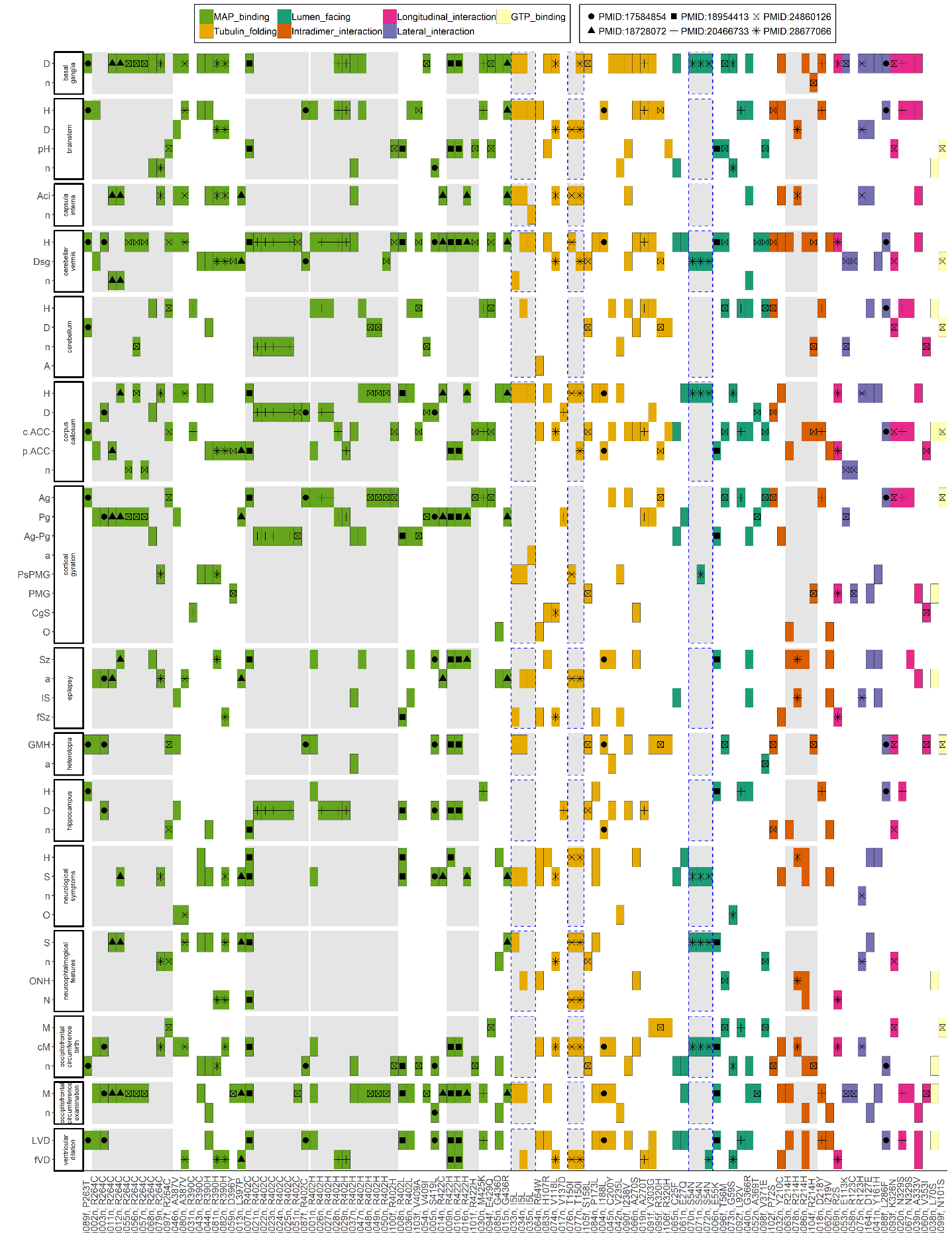
Figure S7 | Matrix plot of all HPO phenotype categories.**

Matrix plot as in Fig. 3A but with all 15 HPO neuroradiological feature groups. Columns represent individuals described here or in the literature. Colors indicate the functional class of the amino acid residue. Symbols indicate the PubMed identifier of publications with ≥ 5 individuals. Individuals and corresponding variants are designated on the x-axis as in Supplementary File S1.


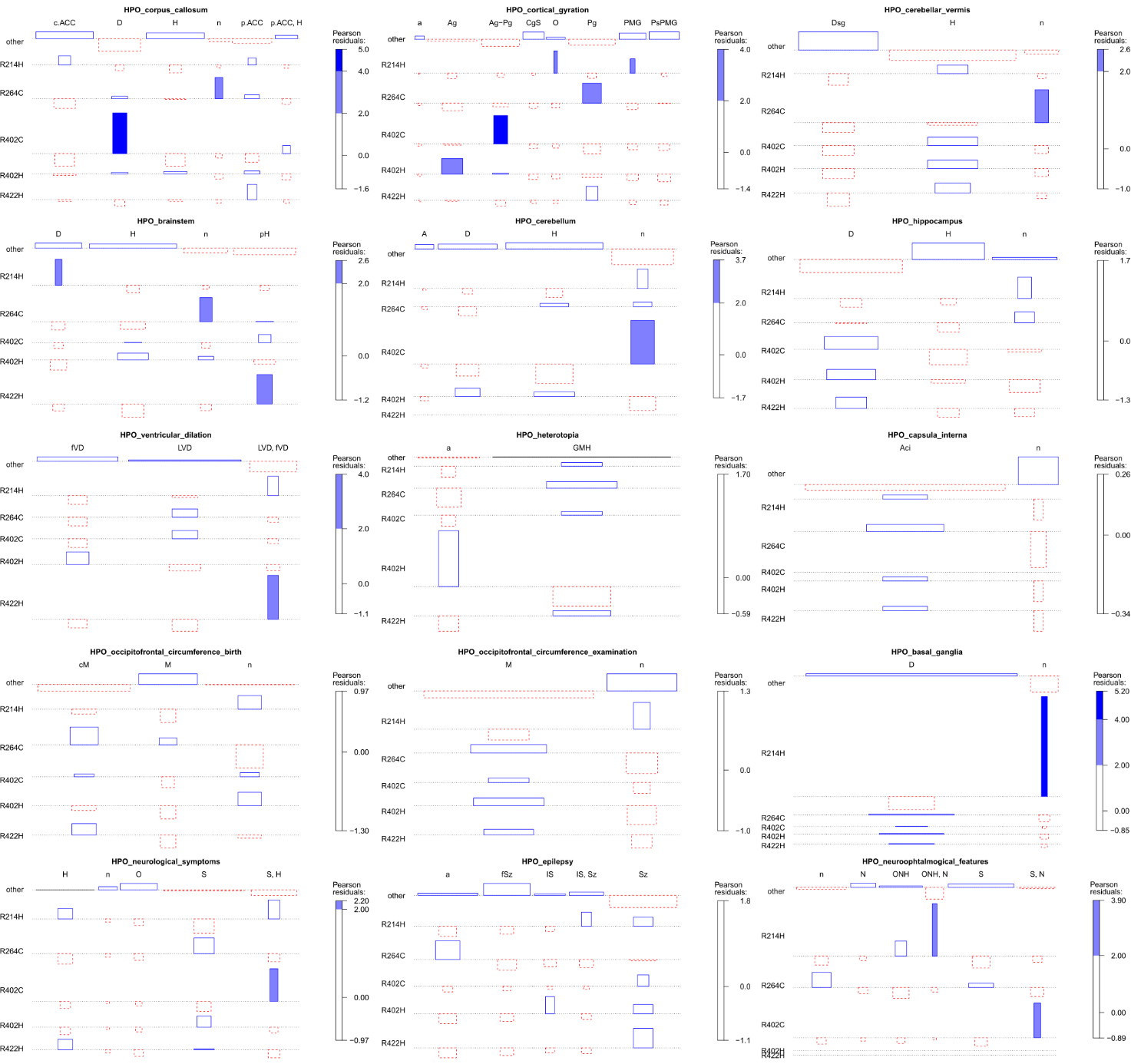


**Figure S8 | Association plots for recurrently affected amino-acid positions and all neuroradiological features.**

All association plots (compare exploratory example in Fig. 3C) showing the relation between recurrent missense variants and all the 15 HPO clinical feature groups. These plots indicate that the clinical features of individuals with recurrent missense variants are not uniformly distributed when compared with the non-recurrent positions, which can be interpreted as a genotype-phenotype correlation. However, there is not enough data to further explore these associations between each variant and feature combination as one would have to correct for multiple testing. Also, for some features which show a trend towards association, there is not enough data show a difference.


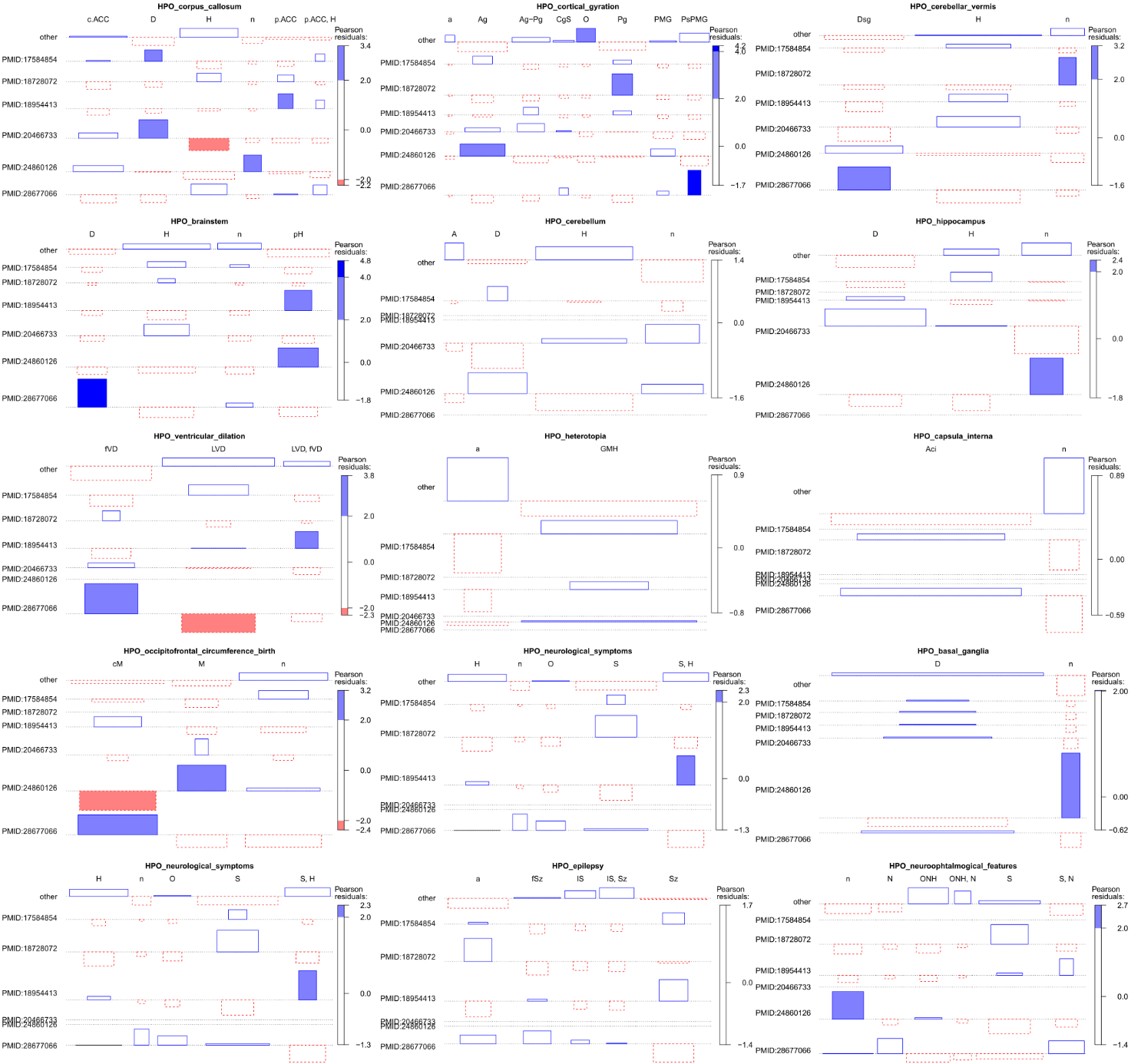


**Figure S9 | Association plots publications describing ≥ 5 individuals and all neuroradiological features.**

All association plots (compare example in Fig. 3D) showing the relation between publications describing ≥ 5 individuals and all the 15 HPO clinical feature groups. These plots indicate a non-uniform use of clinical feature terms in the literature. Also, for some features there is not enough data to show a difference in use.

**TABLES**

| **Table S1 \|** Barthel Index of Activities of Daily Living^3^ of the 3 individuals with *TUBA1A* variants (https://web.archive.org/web/20190110134152/http://www.camapcanada.ca/Barthel.pdf) | | | |
| --- | --- | --- | --- |
|  | **i084n** | **i085n** | **i086n** |
| **Bowels** | 1 | 1 | 0 |
| **Bladder** | 1 | 1 | 0 |
| **Grooming** | 0 | 0 | 0 |
| **Toilet use** | 1 | 1 | 0 |
| **Feeding** | 1 | 1 | 0 |
| **Transfer** | 3 | 2 | 0 |
| **Mobility** | 2 | 3 | 0 |
| **Dressing** | 1 | 1 | 0 |
| **Stairs** | 1 | 1 | 0 |
| **Bathing** | 1 | 0 | 0 |
| **Sum** | 12 | 11 | 0 |

**REFERENCES**

1. Kohler, S. *et al.* The Human Phenotype Ontology in 2017. *Nucleic Acids Res* **45**, D865-D876 (2017).

2. Fisher, R.S. *et al.* Instruction manual for the ILAE 2017 operational classification of seizure types. *Epilepsia* **58**, 531-542 (2017).

3. Collin, C., Wade, D.T., Davies, S. & Horne, V. The Barthel ADL Index: a reliability study. *Int Disabil Stud* **10**, 61-3 (1988).

4. Popp, B. *et al.* Exome Pool-Seq in neurodevelopmental disorders. *Eur J Hum Genet* **25**, 1364-1376 (2017).
